# New employees gain weight in the first 3 years at work: relationship between lifestyle and body weight changes in newly hired male employees in Japan

**DOI:** 10.1093/joccuh/uiaf048

**Published:** 2025-08-19

**Authors:** Masako Yamamura, Yasumasa Matsuba, Kyoko Ito, Hidenori Onishi, Juichi Sato

**Affiliations:** Department of General Medicine, Nagoya University Graduate School of Medicine, Nagoya, Japan; Department of Family Medicine, University of Fukui Hospital, Fukui, Japan; Department of General Medicine, Nagoya University Hospital, Nagoya, Japan; Department of General Medicine, Nagoya University Hospital, Nagoya, Japan; Department of Community Medicine, Faculty of Medical Sciences, University of Fukui, Fukui, Japan; Department of General Medicine, Nagoya University Hospital, Nagoya, Japan

**Keywords:** new employees, weight gain, late dinner, loss of exercise opportunities, work-related stress

## Abstract

**Objectives**: To evaluate lifestyle and weight changes in new male employees of Japanese companies and clarify the effects of environmental and lifestyle changes on weight changes in early years after joining the company.

**Methods**: We analyzed health checkup results and lifestyle questionnaires of 160 male graduates hired by a particular company between fiscal years 2009 and 2012. The data obtained included health examinations from the time of the job offer to the fourth year at the company. Weight changes were analyzed using a Friedman test. Lifestyle questionnaires were analyzed using a McNemar test. Twelve male employees who had been with the company for 5-10 years were interviewed about their lives before and after joining. The results were transcribed and analyzed using the Steps for Coding and Theorization method.

**Results**: Compared with employees’ weight at the time of the job offer, their weight at the time of joining the company and in the second and third years increased significantly. (*P* <.001). An increasing number of participants ate dinner late, missed opportunities for exercise, and did not get sufficient sleep. Interview results indicated that overtime, commuting, and work-related drinking parties among new employees led to late dinners and difficulty in maintaining exercise habits, and that stress at work led to overeating.

**Conclusions**: New employees gained weight during their first 3 years at the company, and lifestyle changes such as overtime work, late dinners due to drinking parties, and loss of opportunities for exercise during the same period had an impact.

## Teaser

- **What we already know about this topic:** In Japan there is much concern about obesity after middle age, and health checkups are conducted annually at companies. On the other hand, there has been little interest in obesity among young people, and few reports have focused on lifestyle changes associated with employment.
- **What this study adds:** The body weight of new graduates increased from the time of receiving an offer of employment to the third year after joining the company. This study modeled the factors and processes that led to the formation of unfavorable eating and exercise habits due to changes in the environment after joining the company.
- **Implications of this study for research, practice, and policy:** The obesity that begins from the time of entry into the company is caused by various lifestyle changes associated with employment, and the company itself needs to improve its structure to eliminate these changes.

## Introduction

Although the average height of young Japanese men has remained approximately the same since the 1980s, their body mass indices (BMIs) have continued to increase in recent years.^1^ There is concern about the higher rates of overweight and obesity in middle-aged and older individuals and an increase in obesity-related diseases.^2^ Against such a background, various workplace health measures have been implemented, including statutory follow-up medical examinations and health counseling; their main purpose is to ensure the safety and proper staffing of companies, although they are also expected to have long-term effects on disease prevention for employees. However, most of these examinations are performed in response to abnormal results of medical examinations, and younger employees without abnormal results are not eligible.

In the Japanese employment system, new graduates are hired all at once.^3^ During the transition period from adolescence to young adulthood, when people of the same generation get jobs all at the same time, this can have a major impact not only on younger people’s independence but also on their sociability, lifestyle, and their views on health. However, little is known about lifestyle changes during this transition period.^4^ Health behaviors are known to be strongly influenced by individual motivation and ability as well as environmental opportunities.^5^ The interaction between psychological stress and dietary patterns leads to obesity among workers.^6^^,^^7^ It is important to acquire knowledge about proper health habits during this period, as they can have a significant impact on future health habits, preferences, and health beliefs.^8^^,^^9^ Several studies have examined factors related to lifestyle habits that contribute to weight gain in young adult males,^10^^,^^11^ although reports evaluating transitions and lifestyle changes are rare.^12^

### Objectives

This study examined the factors of post-employment weight gain among new male employees in Japanese companies by analyzing questionnaires administered during medical examinations and individual interviews.

## Methods

### Study design

To achieve the research objectives, we used a mixed research method. First, we used a quantitative approach to examine factors that contribute to changes in lifestyle and weight gain after joining a company. Next, we used a qualitative research method to analyze the phenomena that led to the results of the quantitative analysis and sought to gain a deeper understanding of the factors involved.

### Quantitative methods

#### Participants

The participants were 315 new male graduates hired by a particular company between fiscal years 2009 and 2012. The business was a trading company with around 3000 employees, and new recruits were assigned to sales or administrative departments as white-collar workers, with their main duties being desk work.

#### Data collection

A total of 160 participants had their weight and interview data available at the time of the job offer, on joining the company, and at health checkups in the second, third, and fourth years after joining the company. The questionnaire consisted of 2 dietary questions: “Do you eat dinner within 2 hours of going to bed at least 3 times a week?” and “Do you skip breakfast at least 3 times a week?” The 2 physical activity questions were: “Have you completed light sweat-inducing exercise for at least 30 minutes twice or more per week for more than 1 year?” and “Do you walk or do equivalent physical activity for at least 1 hour daily?” The 1 sleep question was: “Do you get enough rest through sleep?”

#### Data analysis

Statistical analyses were performed using EZR version 1.54 (Saitama Medical Center, Jichi Medical University, Japan).^13^ Continuous variables are reported as mean ± SD, whereas nominal variables are presented as counts and percentages. Weight changes after joining the company were analyzed using a Friedman test (multiple comparisons of 2 groups at a time with Bonferroni adjustment) because we expected large individual differences in the multi-group data and problems with normality. The significance level for all tests was set at .05. To compare the proportion of participants with specific lifestyle habits at the time of job offer and at follow-up, a McNemar test was used. This test was chosen because it is appropriate for analyzing paired nominal data from the same individuals over time. The significance level for all tests was set at .05.

### Qualitative methods

#### Participants

Participants were those who had periods of weight gain after joining the company, as identified through health interviews conducted when they reached milestone ages in 5-year increments. These 12 employees agreed to be interviewed and have the interview results used for research. Table 2 shows the participants’ work details.

#### Data collection

One-on-one semistructured interviews were conducted with 12 male employees who joined the company after 2009 and worked for 5-10 years. The interviews lasted for approximately 20-30 minutes (see Supplementary Material). The interviews were recorded and transcribed verbatim.

#### Data analysis

Transcripts were qualitatively analyzed using the Steps for Coding and Theorization method.^14^^,^^15^ It consists of the following steps: (1) Identify noteworthy words or phrases from the text. (2) Rephrase the words and phrases extracted in the previous step. (3) Create concepts that explain the words listed in the previous step. (4) Create themes and constructs considering the context in the third step and writing theories from the storyline. The interviewer segmented the text, extracted keywords, and wrote down the themes and conceptual ideas. To ensure validity, the themes and conceptual ideas were checked by 2 researchers (M.Y. and J.S.), after which the storyline was created and the logical description was written.

This study was conducted with the approval of the Bioethics Review Board of the Nagoya University Graduate School of Medicine (approval number: 2018-0135).

## Results

### Quantitative results

**Figure 1:**
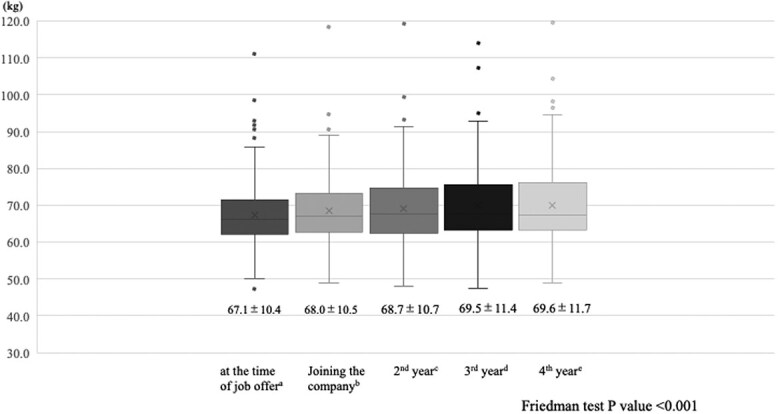
*P* value adjustment method: Bonferroni. a vs b *P* value <.001, a vs c *P* value <.001, a vs d *P* value <.001, a vs e *P* value <.001, b vs c *P* value = .002, b vs d *P* value <.001, b vs e *P* value <.001, c vs d *P* value <.001, c vs e *P* value = .002, d vs e *P* value = 1.000.

The mean interval between the medical examination at the time of the job offer and employment was 248 days. The mean age at the job offer was 22.8 ± 1.3 years, the mean body weight was 67.5 ± 9.5 kg, and the mean BMI was 22.6 ± 2.7 kg/m^2^. Compared with the weight at the time of the job offer, the weight increased significantly at the time of joining the company and in the second, third, and fourth years (Figure 1). Significant differences were observed in all combinations except for the combination of third and fourth years of employment (*P* < .001).

The details of the questionnaire are shown in Table 1. Compared with the time of hiring, the percentage of employees who answered “yes” to the question about skipping breakfast decreased significantly after joining the company (47% vs 16%, *P* < .001). There was no significant difference in the percentage of employees who had the habit of eating dinner late. On the other hand, the proportion of employees who answered “no” to questions regarding “exercise habits,” “physical activity,” and “enough rest through sleep” increased (*P* < .001). When comparing at the time of job offer with 4 years after employment, no changes were observed in the habit of skipping breakfast. Furthermore, the proportion of unhealthy habits increased significantly in all other questions (*P* < .001).

**Table 1:** Lifestyle percentages from time of job offer to post-employment periods (*n* = 160).^a^

	**Late dinner**	**Skipping breakfast**	**Exercise habits**	**Physical activity**	**Enough rest through sleep**
At the time of job offer	41 (25)	76 (47)	85 (53)	114 (71)	146 (91)
Joining the company	57 (35)	26 (16)	47 (29)	82 (51)	101 (63)
Second year in the company	111 (69)	61 (38)	45 (28)	55 (34)	89 (55)
Third year in the company	111 (69)	73 (45)	39 (24)	60 (37)	98 (61)
Fourth year in the company	103 (64)	77 (48)	39 (24)	66 (41)	101 (63)

a All values are *n* (%).

**Table 2:** Work contents of 12 participants.

**Participant ID**	**Work content**
A	Textiles, sales
B	Logistics planning, planning
C	Material recycling, sales
D	Sales development, sales
E	Logistics trade, management
F	Electronic materials, sales
G	IT strategy, sales
H	Electronic devices, sales
I	Human resources, recruitment, management
J	Resins, chemicals sales, sales
K	IT management, information industry/sales
L	Industrial materials, sales

### Qualitative results

From the results of these individual interviews, patterns of lifestyle changes that occurred before and after starting work and the accompanying weight gain were extracted, and a story was derived. Three stages were found in the flow of changes leading to weight gain: environmental changes (such as changes in working hours and place of work due to starting work), which affected eating and physical activity, and the establishment of an undesirable lifestyle.

#### Environmental changes due to employment

As part of the environmental changes associated with employment, employees were also required to follow the chain of command in the organization based on a performance-based evaluation system from the very beginning of their employment.

*From the first to the fifth year of employment, employees had almost no right of veto as a member of the company.* Employee B.

These changes had various effects on lifestyle habits such as eating and exercise habits.

#### Restrictions on eating behavior

Opportunities to eat out and drink alcohol for communication purposes about work increased, and the habit of drinking alcohol daily had been established. There was a culture of going out for drinks with colleagues after work, which was semi-compulsory and sometimes lasted until late at night.

*I join them even though I’m full.* Employee K.

#### Abnormalities in eating behavior that lead to obesity

Due to late-night overtime work, dinner was delayed, and there were more opportunities to eat out with high-calorie meals.

*Since I mostly eat out, I end up eating a lot of fatty foods.* Employee G Consuming sugar to stave off hunger while working overtime was recognized as an unhealthy habit that could not be avoided.

*When I’m too busy and my brain starts to slow down, I drink sugary energy drinks or eat chocolate.* Employee B.

Sleep also became irregular, and the amount of sleep decreased. For example, on business trips, one participant continued to eat out from a limited menu of greasy meals. On overseas business trips, business associates sometimes met for meals every day, including weekends. At dinner parties, participants found it difficult to refuse offers of food or alcohol from superiors, and their weight increased weekly.

*When I go on business trips, I often eat high-calorie foods during the day, and the expatriates give me drinks at night, so I gained 4 kg in about 2 weeks.* Employee B.

*Our customers also like alcohol, and our business is based on drinking and doing business.* Employee L.

#### Restriction on leisure time activities

Immediately after joining the company, the new employees had relatively free time, especially during the training period, and they maintained their exercise habits by going to the gymnasium to lose weight. A process was found whereby increased workload and communication through food gradually made it more difficult to find leisure time.

*When I was a new employee, I lived in a dormitory with a workout room. However, as the workload and time spent at work increased, the rhythm of daily life became irregular, and around the third year, the time to the last train home increased, and exercise habits ceased.* Employee B.

#### Loss of exercise opportunities

The loss of leisure time led to the loss of exercise places, exercise opportunities, exercise companions, and motivation to exercise, which in turn led to the loss of acquired exercise habits.

*During my third and fourth years in the company, I was so busy that I took the last train home every day. At that time I could not exercise. I slept at home all Saturday and Sunday.* Employee B.

It was recognized that a moderate workload was important for maintaining good eating and exercise habits.

*After joining the company, there were times when I led a healthy lifestyle, and then there were times when my health suddenly became unhealthy. I think I had the most time to exercise when I had a moderate workload.* Employee B.

When overeating and lack of exercise reached a certain point, weight increased.

#### Factors that worked to maintain weight

Those who did not gain weight after joining the company maintained the following desirable eating and exercise habits.

*I stepped on the scale every day, paid attention to changes in my clothing size and body shape, followed a low-carbohydrate diet, and increased my opportunities for exercise.* Employee C.

*I developed the habit of exercising since childhood and continued to run once or twice a week even after joining the company.* Employee E.

*I developed the habit of exercising by participating in a relay race held in the company.* Employee F.

## Discussion

The prevalence of adult obesity worldwide has doubled since 1990, and that of adolescent obesity has quadrupled.^16^ Although the obesity rate among adult Japanese men is not high compared with other countries,^17^ the body weight of Japanese people increases during their 20s and 30s.^18^^-^^20^ The main risk factors for overweight and obesity are an increase in eating out, poor diet quality (increased consumption of sugary drinks, snacks, and fast food), and decreased physical activity associated with typical adult life events such as employment and marriage.^21^ Once someone becomes obese, it is not easy to return to normal weight.^22^ In addition, severe obesity during adolescence leads to increased morbidity and mortality from other diseases, including coronary heart disease, during prime working age.^2^ Therefore, weight control at an early age is important for reducing this risk.

Although weight gradually increased from the time of the job offer to the first, second, third, and fourth years after joining the company, there were different patterns of change in lifestyle habits related to obesity. Between the time of receiving a job offer and the time of joining the company, a significant percentage of participants reported decreases in exercise habits, physical activity levels, and getting enough rest through sleep.

Regarding exercise habits, more than half of Japanese men in their 20s exercise or play sports at least once a week,^23^ as did the majority of participants in this study at the time of the job offer. Opportunities for physical activity may have decreased as students graduated from university sports clubs. The decrease in physical activity may have been due to factors such as fewer opportunities to commute to school and play with friends.

The percentage of Japanese men in their 20s who have reported that they get enough rest through sleep is just under 80%^24^; however, among the participants in this study, this percentage dropped from 90% at the time of the job offer to 60% at the time of joining the company. This result suggests that the amount of sleep decreases when people begin working.

The percentage of participants who ate dinner late increased after the second year of joining the company. This is because when employees first join the company, there is an orientation period during which overtime work is minimal, whereas in the second year overtime work and entertainment opportunities increase.

Regarding breakfast habits, the breakfast consumption rate for Japanese men in their early 20s is more than 60%^25^; the rates among the participants in this study at the time of the job offer were similar. Among the participants the percentage of those who did not eat breakfast was 47% at the time of the job offer, but it dropped significantly to 16% at the time of joining the company, then returned to the original level in the second year of employment. The reason for this phenomenon is that many new employees live in company dormitories. The interviews revealed that this reflected a disciplined lifestyle during the training period, and it is possible that the employees in the dormitory ate breakfast properly when they first entered the company but stopped eating breakfast when they left the dormitory. These results suggest that maintaining the habit of eating breakfast is difficult.

This study showed that quantitative surveys of health checkups and questionnaires revealed that new employees gained weight early after joining the company, and that the percentage of people whose lifestyle habits worsened during the same period increased. However, although a weak correlation was found between late dinner habits and weight gain, quantitative analysis alone did not reveal a significant relationship between lifestyle changes and weight gain. We conducted the interview because we thought that it would be difficult to clarify the actual lifestyle by only conducting a questionnaire survey. As a result, qualitative analysis of interviews revealed the detailed process by which changes in the environment after joining the company led to lifestyle changes that could result in weight gain.

**Figure 2:**
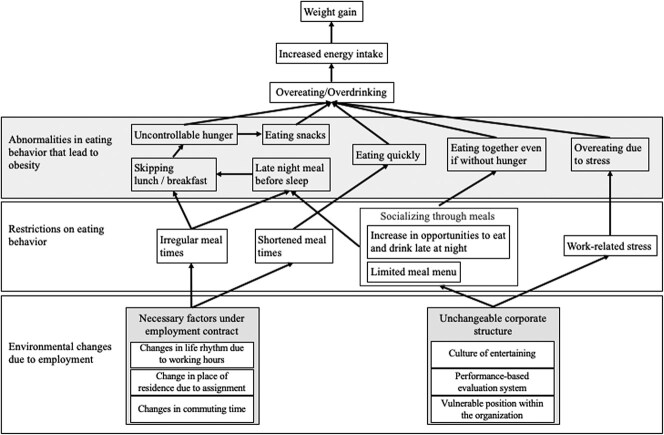
Changes in eating behavior resulting in weight gain due to environmental alterations from employment.

**Figure 3:**
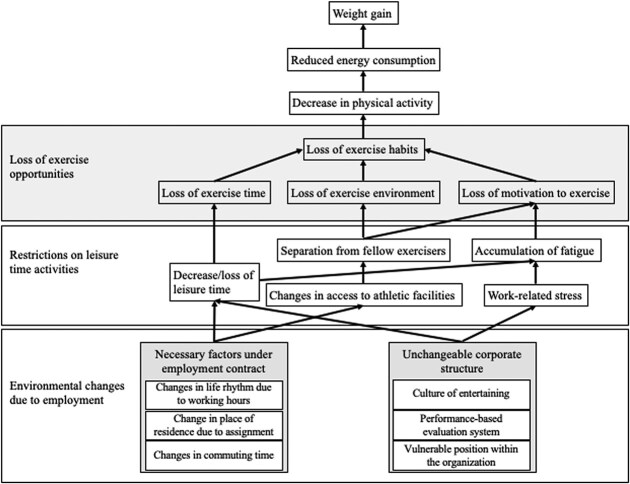
Changes in exercise behavior resulting in weight gain due to environmental alterations from employment.

From the analysis of the individual interviews, we created conceptual diagrams that focus on the environmental changes leading to behavioral changes causing weight gain after joining the company (Figures 2 and 3).

Concerning eating behaviors, new employees face unavoidable alterations under their employment contracts, such as changes in their daily rhythms upon starting work, place of residence due to their assignments, and commuting time. In addition, the company in this study has characteristics such as a culture of customer service and performance-based evaluation systems. New employees are in weak positions in a vertically structured society with strict hierarchical relationships; therefore, they find it difficult to refuse their superiors. Due to the changes in the environment that come with starting work, mealtimes become irregular and shorter. Irregular mealtimes can lead to eating late in the evening before going to bed and skipping breakfast or lunch, which can produce feelings of hunger and a propensity for snacking. Additionally, when mealtimes become shorter, people tend to eat faster. When entertaining with a meal to facilitate working relationships, mealtimes tend to be delayed. Eating a balanced diet in the context of work-related entertainment is difficult because of limited menu choices. During social meals, people eat even when they are not hungry. These abnormal eating behaviors can lead to overeating and binge eating. Unchangeable corporate structures prevalent in the industry, such as doing business over drinks with business partners, create stress that leads to overeating, which results in increased energy intake and weight gain.

Regarding physical activity, owing to changes in the environment caused by the start of work, leisure time is reduced or lost, and it becomes difficult to attend exercise facilities (Figure 3). Reduced or lost leisure time and work-related stress caused by industrial customs can lead to chronic fatigue. Difficulty in getting to an exercise facility causes separation from fellow exercisers, resulting in the loss of an exercise environment and the motivation to exercise. The accumulation of fatigue also can result in the loss of motivation to exercise. When the time, environment, and motivation to exercise are lost, exercise habits disappear. As a result, physical activity and energy expenditure decrease, and weight gain occurs.

Emerging adulthood is a new concept in human development that corresponds to the period from late teens to twenties, and habits formed during this period tend to persist throughout life.^26^ This is considered an important period for the prevention and treatment of obesity, and it coincides with the period when the participants of this study gained weight before and after joining the company. Therefore, it is important to intervene to prevent lifestyle-related diseases among young adults in their 20s, who are in the prime of their working lives, rather than waiting until they become obese in their 40s.

As with many diseases, the causes of obesity are not simply a matter of personal will, but a complex interaction of multiple factors.^27^ It is important to improve the health literacy of individuals and take actions that focus on environmental factors specific to groups, including psychosocial factors, rather than focusing only on individual motivation and health education.^28^

This study found that a company’s entertainment policy has a significant impact on new employees’ eating, exercising, and sleeping habits. Overeating, lack of exercise, and sleep deprivation are associated with weight gain.^29^ Chronic work-related stress can lead to weight gain^30^ and increase the risk of developing metabolic syndrome.^6^^,^^7^ A results-oriented evaluation system is not necessarily bad; however, if taken too far, it could lead to employees compromising their health to achieve results. In recent years, overwork and strict hierarchical relationships have been considered problems of power harassment. However, by addressing this issue, companies can increase employee leisure time and reduce work-related stress, which can lead to improved eating and exercise habits.

The Centers for Disease Control and Prevention’s Workplace Health Model recommends workplace programs aimed at improving diet and physical activity behaviors to help employees lose weight. Workplace health programs (WHPs) include various approaches to support behavior change.^31^ However, cost, providers, and a lack of management support are barriers to WHP implementation.^32^ In addition, psychosocial factors, workplace organization, and the work environment can have a significant impact on the success or failure of a WHP.^33^ In interviews for this study, participants described the loss of good health habits due to an environment that made it difficult to maintain a healthy lifestyle. Barriers should be removed at the organizational level to address work–life balance constraints that prevent healthy lifestyles.

### Limitations

There are a few limitations of this study. First, the participants were limited to a single company, which limits the ability to generalize weight gain and work-related lifestyle changes among new employees. Second, the interviews were conducted more than 5 years after the employees joined the company, thus there is a time lag, and their recollections may have been vague. Finally, this survey was conducted before work style reform was implemented as a national policy in Japan and may not have fully reflected recent changes in corporate attitudes toward overtime.

## Conclusions

New employees gained weight by their fourth year of employment. Individual interviews indicated that in addition to essential factors related to employment, such as changes in eating and exercise habits caused by working hours, residence relocation, commuting time, corporate customs such as a culture of entertainment, and work-related stress due to results-oriented evaluation systems were the causes of weight gain after joining the company.

## Supplementary Material

Web_Material_uiaf048

## Data Availability

The data are not publicly available due to the inclusion of information that may infringe on the privacy of research participants.
