# Supplementary material for: New employees gain weight in the first 3 years at work: relationship between lifestyle and body weight changes in newly hired male employees in Japan
Source: J Occup Health. 2025 Aug 19;67(1):uiaf048. doi: 10.1093/joccuh/uiaf048 (PMC12481241; doi:10.1093/joccuh/uiaf048)
Supplement: Web_Material_uiaf048 [file web_material_uiaf048.zip › Web_Material_uiaf048.docx]

Appendix 1 ; An outline of the interview guide and questions

**Current perception of body**

・What is your current weight?

・What was your weight when you joined the company?

・What do you think is a suitable weight?

Please tell us the reason why you think that weight is suitable.

**Life before joining the company**

・Did you have any regular opportunities or habits of exercise?

(club activities, part-time work, etc.)

・ What kind of work have you been in charge of since joining the company?

**Lifestyle changes and their causes**

・What kind of lifestyle changes have you observed after joining the company?

・What are the causes of these changes? (work-related/private)

-Type of work/duties

- Living environment

Living alone, living with parents, living in a dormitory, marriage, living together, etc.

- Physical activity

Commuting time to and from work, walking time, daily activities, etc.

- Eating habits

(Whether or not you have breakfast, the time you eat dinner, regularity, changes in the time and amount of food you eat, feeling of fullness, food content, amount of alcohol consumed, frequency of alcohol consumption, etc.)

- Exercise habits

Whether or not you have regular exercise habits, exercise intensity, etc.

- Sleep habits

The time you go to bed and wake up, quality of sleep, etc.

**Influence of work life on body weight**

・What factors have affected your lifestyle since joining the company? Please tell us about both positive and negative influences.

(work content, overtime, business trips, meals with clients, transfers, etc.)

**Influence from other people**

・Has anyone influenced your lifestyle/life style since joining the company?

Please tell us about both positive and negative influences.

(work style, exercise habits, drinking, smoking habits, etc.)

**Background to stress**

・Please tell us about the stress you have experienced at work.

・How did this stress affect your lifestyle/habits?

(diet, drinking, physical activity, exercise habits, sleeping hours, etc.)

**Weight change and its causes**

・What is your heaviest weight in your life? When was that?

Please tell us the background to that.

・What was the trigger for losing weight/dieting?

・What were the causes of weight gain/loss after joining the company?

**Personal health awareness**

(weight maintenance methods/interpretations)

・Are there any behaviours or habits you try to maintain your weight?

・Are there any new habits you have adopted since joining the company?

・What do you think are the behaviours and habits that are effective for maintaining your weight?

・What do you think are the reasons you are able to maintain your current weight?

**Perception of changes in lifestyle**

Habits

・Has your lifestyle become healthier or less healthy since joining the company?

**Other**

Leisure activities (changes in the length of leisure time and how you spend it)
